# A feasibility study on the use of phantoms with statistical lung masses for determining the uncertainty in the dose absorbed by the lung from broad beams of incident photons and neutrons

**DOI:** 10.1093/jrr/rrw118

**Published:** 2017-01-11

**Authors:** Atiyeh Ebrahimi Khankook, Hashem Miri Hakimabad, Laleh Rafat Motavalli

**Affiliations:** 1 Physics Department, Faculty of Sciences, Ferdowsi University of Mashhad, Mashhad 91775-1436, Iran

**Keywords:** statistical phantom, lung-absorbed dose, broad parallel beams, MCNP5, mono-energetic photon and neutron exposures

## Abstract

Computational models of the human body have gradually become crucial in the evaluation of doses absorbed by organs. However, individuals may differ considerably in terms of organ size and shape. In this study, the authors sought to determine the energy-dependent standard deviations due to lung size of the dose absorbed by the lung during external photon and neutron beam exposures. One hundred lungs with different masses were prepared and located in an adult male International Commission on Radiological Protection (ICRP) reference phantom. Calculations were performed using the Monte Carlo N-particle code version 5 (MCNP5). Variation in the lung mass caused great uncertainty: ~90% for low-energy broad parallel photon beams. However, for high-energy photons, the lung-absorbed dose dependency on the anatomical variation was reduced to <1%. In addition, the results obtained indicated that the discrepancy in the lung-absorbed dose varied from 0.6% to 8% for neutron beam exposure. Consequently, the relationship between absorbed dose and organ volume was found to be significant for low-energy photon sources, whereas for higher energy photon sources the organ-absorbed dose was independent of the organ volume. In the case of neutron beam exposure, the maximum discrepancy (of 8%) occurred in the energy range between 0.1 and 5 MeV.

## INTRODUCTION

Radiation dosimetry is an essential aspect of occupational radiation protection as well as of diagnostic medical procedures and therapeutic techniques. With the advent of Monte Carlo calculations and the development of computational phantoms (hereafter abbreviated to phantoms), the feasibility of simulation and estimation of the dose absorbed by various organs has been explored. Using these anatomical models, organ-absorbed and effective doses have been obtained for various ionizing radiation fields.

To minimize radiation-induced deterministic damages and to protect against occupational exposures, the International Commission on Radiological Protection (ICRP) has set dose limits for some sensitive organs (such as the eye), and also for effective dose [1–3]. Dose limitations are calculated using a reference phantom that represents an individual at the 50th height/weight percentile in a given human population [4–9]. Also, for determining the organ-absorbed and effective doses in diagnostic techniques, such as modern computed tomography (CT) or *in vivo* neutron activation analysis (IVNAA), and estimating delivered dose from secondary radiation to organs away from the target volume in radiotherapy, a reference phantom can be a very profitable instrument [10–13].

In spite of many advantages of reference phantoms for developing dose coefficients for radiological protection and occupational exposures, their application is limited, especially when an individual has a body morphometry far from the 50th height/weight percentile [14]. In fact, reference phantoms can only be used for estimation of the dose conversion coefficients of an ‘average’ individual; they cannot be applied for individuals with different anatomical data.

Investigators have coped somewhat with the limitations of using reference phantoms by making a patient-dependent series of five height/weight percentiles using hybrid phantoms. In hybrid computational phantoms, organ boundaries are described by combinations of polygon mesh or Non-Uniform Rational B-Spline (NURBS) surfaces [14]. Therefore, different height/weight percentiles can be made by modifying a reference model around the anthropometric distributions of the adult population.

To date several reports have described the influence of body size on dose conversion coefficients using patient-dependent phantoms. Johnson *et al*. studied the influence of patient size on dose conversion coefficients for a representative interventional fluoroscopic procedure in 2009 and showed the need for using diverse anatomical models for medical dosimetry [15]. In 2012, Ding *et al*. also examined the effect of obesity on the calculated radiation dose for organs and tissues in CT scans [10]. In addition, according to the report by Bolch *et al*. there was a critical need to update the databases that had been provided using current age-matched 50th percentile reference phantoms in modern CT imaging [13]. This was an intermediate solution to match the height/weight of a specific patient phantom to the target height/weight percentiles; however, the resultant phantoms would be dependent on the primary patient anatomy.

In late 2009, the Taylor and Francis Group published a handbook of anatomical models for radiation dosimetry [13]. This comprehensive book is a collection of some interesting and important literature about the construction of various types of human phantoms and their medical and industrial applications, and it can serve as a reference book as well as a guide for the evolution of human anatomical models for radiation dosimetry. The present work investigates the concerns raised in that book about the difficulties in creating a specific phantom and in determining the uncertainty of the estimated dose as assessed by reference phantoms.

Currently, there are many reference phantoms developed by various groups, and some sets of patient-dependent phantoms. The organ dose levels obtained by these phantoms can differ significantly, because their initial anatomies have been based on different people. The major issue is: what is the level of uncertainty in organ doses for a group of people, as determined from reference models?

In the Third International Workshop on Computational Phantoms for Radiation Protection, Imaging and Radiotherapy, held August 2011 in China, a creative new method was suggested for determining the sizes of doses and dose uncertainties at the same time [16]. According to that proposal, it may be possible to determine the uncertainty in the organ-absorbed dose due to the organ shape, mass and position by changing the organ, based on the statistical distribution of anatomical variations. This approach helps to identify the accuracy of dose data and the necessity of creation of a patient-specific phantom for the specific irradiation. To date, several studies have been performed on the creation of deformable phantoms based on the statistical distribution to cover a greater range of body morphometry than the reference phantom [17, 18].

In 2010, Babapour *et al*. presented a computational framework based on statistical shape modeling for construction of race-specific organ models [17]. They applied a spherical harmonics shape descriptor on a Japanese liver phantom without replacing the resultant models in a whole-body phantom.

Recently, Segars *et al*. from Duke University Medical Center extended the XCAT phantom to provide the first library of 4D computational phantoms, by developing a series of anatomically variable 4D XCAT adult phantoms for imaging research [18].

The aim of the present work was to investigate the effect of variation of lung volume on the lung-absorbed dose per fluence (LADF). This could be a primary study for investigating the applicability of phantoms with statistical deformable lung for dosimetry calculations when the lung mass (size) varies based on a normal distribution. The data obtained from this phantom series could be used to provide an accurate and valid dose library, which would report the average organ-absorbed doses and their deviations.

The adult male (AM) ICRP reference phantom was considered as a basic model [19]. Commencing with the basic model, 100 phantoms were created with different lung sizes. The phantoms with statistical lung were irradiated by the standard irradiation geometries of mono-energetic photons and neutrons, and then dose uncertainties as a result of the lung size variation were calculated. The results of this work might provide a clue for construction of statistical whole-body phantoms for defining the conversion coefficient uncertainties of the major organs, especially in diagnostic systems such as fluoroscopy, CT scanning or IVNAA.

## MATERIALS AND METHODS

There are some parameters that may affect the lung dose. Some of the important parameters are the size, shape and position of the lung; the size, shape and position of the other internal organs, and also whole-body frame properties such as trunk shape and location of arms. In order to investigate the lung size effect on the LADF, other effective parameters were minimized by considering some simplifying assumptions. For example, the shape and position of the lung were fixed during the lung size variation. Moreover, a whole-body phantom was considered as a template model for constructing a phantom series. Each phantom of the series was created by extracting the lung of the basic phantom, resizing it to desired volume and replacing it in the same position as that of the original lung in the whole-body frame. The validity of the assumptions and a detailed description of the phantom construction are discussed in the first section. Moreover, some brief discussions about the distribution of the lung size, statistical parameters, which are used to illustrate the LADF uncertainty (uncertainty parameters), and Monte Carlo simulation details are provided in the following sections.

### Phantom construction

#### The validity of using a template phantom

Whole-body properties such as the trunk shape, location of arms, and length of legs could be very important in external dosimetry. For example, the location of arms may affect the lung dose in lateral irradiation geometries. For minimizing the effects of these parameters on obtained LADFs and in order to provide similar conditions for all constructed phantoms, the lung models were imported into a template whole-body phantom.

**Fig. 1.**
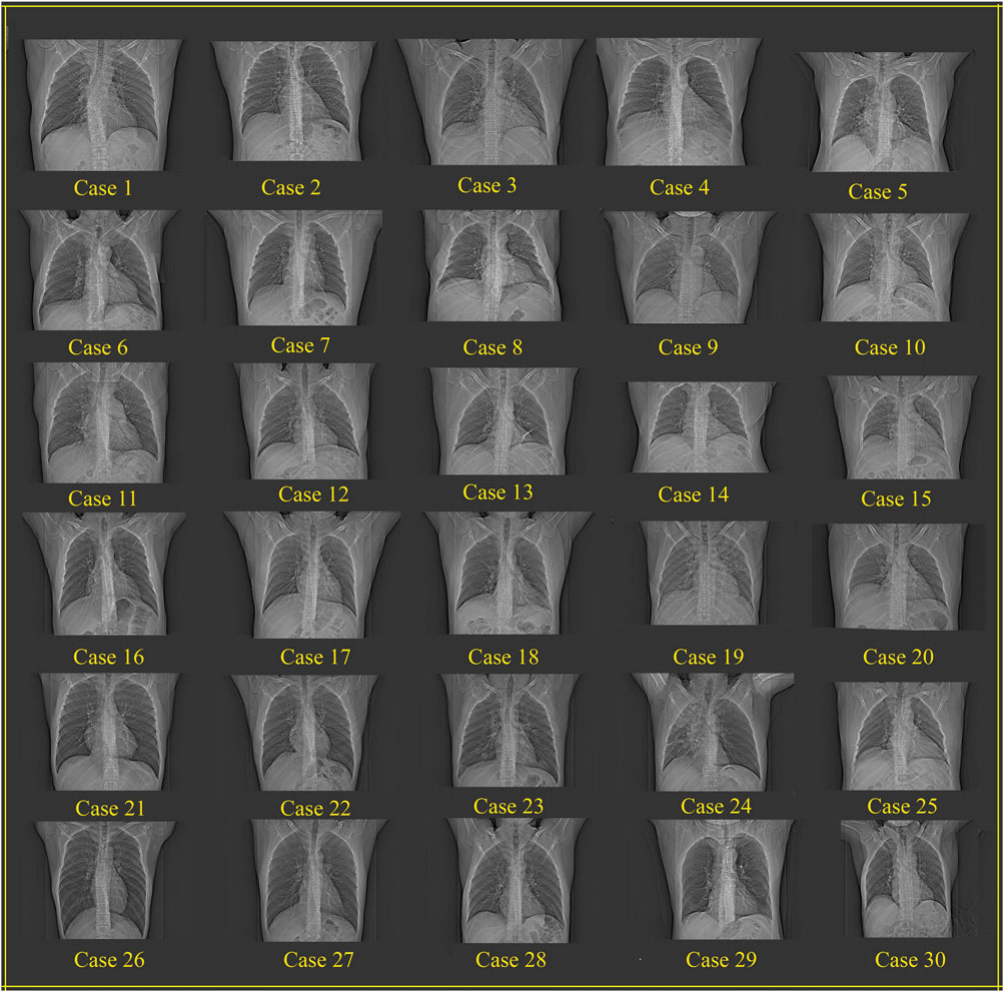
Comparison of the lung size of 30 normal patients in radiography images. The cases that have been placed in a particular row belong to the same height/weight percentile. The variation in lung volume for the patients in any given row is clear.

To check that people with a similar whole-body frame may have different lung sizes, the anatomical data for a number of individuals were determined from medical images obtained from a database of the Parsian Medical Imaging Center of Mashhad. Studying chest CT and radiography data from patients with normal lungs showed that the lung sizes of individuals, who belong to the same percentile of height/weight could differ. Figure 1 shows the radiography images of 30 patients. In this figure, the cases shown in the same rows belong to the same height/weight percentiles. Ignoring very small differences in the X-ray images, which are due to the small changes in field of view (FOV) or magnifications, it is clear that the lung sizes of cases located at the same row vary considerably. Thus, using a whole-body frame for constructing a series of phantoms with different lung sizes would be an acceptable assumption.

#### The characteristics of a template phantom

The ICRP reference AM phantom, which was considered as the template model in this work, is based on the medical image data of real people with anatomical and physiological parameters consistent with the reference information given in ICRP Publication 89 [20]. The voxel size of the AM phantom was 8 mm in height, with an in-plane resolution of 2.137 mm, resulting in ~1.95 million tissue voxels [19]. In this investigation, the lung mass (size) was considered as the statistical parameter. Hence, characteristics of the ICRP lung are discussed in more detail. In both ICRP 89 and ICRP 110, the reported reference value for the mass of the adult male lung is equal to 1200 g [19, 20]. However, considering the mass data given in Table A1 of ICRP Publication 110 for the masses of tissue and blood of the right and left lung lobes, the whole mass of the ICRP lung is 1208.37 g [19]. This value is actually very consistent with the reference mass that reported by the Reference Man task group of ICRP in ICRP Publication 89 [20].

Table 1 indicates the mass and density data of the tissue and blood of the right and left lung lobes, which was provided in ICRP Publication 110 [19]. ICRP Publication 89 reported the specific gravities (the substance density divided by pure water density) of adult lungs either free of air but with the blood vessels completely filled free of air, containing air, and free of air and blood [19]. However, in the ICRP AM phantom, the specific gravity of the compressed lungs was considered as the lung tissue density, which is higher than that of the lung containing air and lower than that of the lung with blood-filled vessels. In the present work, to make LADFs comparable with dose data provided with ICRP Publication 110, the density of the compressed lung was used.

**Table 1.** The mass and density of each part of the adult male lung [20]

Tissues	Mass (g)		Density (g/cm^3^)
	Right lobe	Left lobe	
Lung tissue	580.08	477.74	0.382^a^
Blood	71.54	79.01	1.06
Total	651.62	556.75	-
**Total lung mass**		**1208.37**	

^a^The density of the compressed lung reported in ICRP Publication 110 is considered.

According to the data given in Table 1, the lung volume can be assumed to be ~2911.190 cm^3^. The right lobe has a higher volume, total capacity and mass than the left lobe, because the heart is mostly on the left side of the body. The total volumes of the right and left lung lobes are 1586.02 cm^3^ and 1325.17 cm^3^, respectively. The lung position can significantly influence the dose conversion coefficients from external exposures because of the attenuation properties of the tissues surrounded the lung volume. In ICRP Publication 110, the *x*, *y* and *z* positions of the centroids of the right and left lung lobes were reported (20.92 cm, 14.79 cm, 137.1 cm) and (34.86 cm, 15.8 cm, 137.2 cm), respectively [19]. In this work, to reduce the influence of the lung position on the lung dose, the right and left centroids were fixed at these points.

#### The modifications of the lung and other internal organs

The Modification of the voxel model of the AM lung and its adjustment to the desired volume was a hard and sophisticated task because changing a voxel model would be limited to the voxel size in different dimensions. Therefore, 3D-Doctor^TM^ software (version 4; Able Software, Corp, Lexington, MA) was used to create a 3D model of the AM lung and to convert voxel geometry into a polygon mesh form. The resultant model was rescaled and matched to a new volume and was also revoxelized by the software program Rhinoceros^TM^ 5.0 (version 5, Robert McNeel Assoc.). Detailed information about voxelization using Rhinoceros 5.0 has been provided by Hoseinian-Azghadi *et al*. [21].

In addition to the lung, the mesh model of the internal tissue (lung vessels) was also extracted from voxel geometry. The volume of the blood vessels was changed proportionally to the variation in the related lung volume. In order to investigate the correlation between the lung and vessel volumes, some models and their vessel trees were segmented and extracted from the existing CT images. According to the segmented models, the size of the vessel tree changed proportionally to the lung size, whereas the thickness of the vessels did not vary with the lung size. Thus, to construct statistical lungs, the final volume of the vessel trees was determined by multiplication of the initial volume by the same factor of the related lung. Changing the vessel tree volume, the main tree remained unchanged, and only a number of branches were altered. In the case of large lungs, some vessel branches were added to vessel-less regions, which were created due to the lung inflation. For small lungs, the branches located outside the lung were eliminated. Moreover, the lengths of some branches were truncated in high concentration regions, so that the desired vessel volume was achieved.

For the proper insertion of modified models of the lung and vessels into the phantom, the voxels of the primary models were eliminated and then new voxels were imported into the 3D matrix. In the ICRP phantom, the lung and its adjacent organs (such as rib cage, heart, and liver) were separated by a few millimeters of trunk muscle and residual tissue. Therefore, for phantoms with small lungs, the residual tissue filled the voxels, which remained empty during lung deflation.

**Fig. 2.**
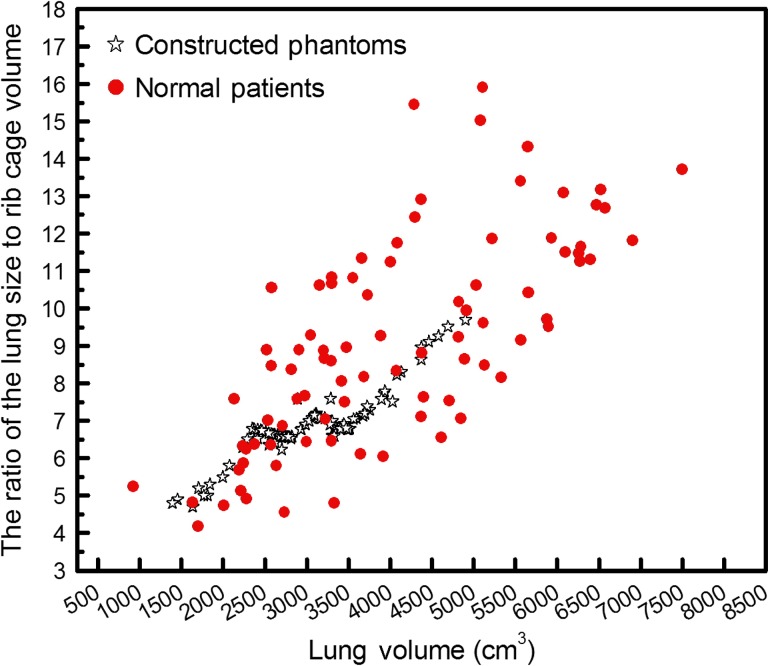
This scatter plot shows the correlation between lung size and rib cage volume for normal patients and constructed phantoms.

On the other hand, inflating the lung to increase the volume caused some overlaps between the lung and its adjacent organs. In the worst situation, the largest lung covered nearly 70% of ribs, 10% of the heart and 8% of the liver. In fact, the size and shape of the lung and rib cage are strongly related to each other. Moreover, due to the high density of bone tissue, the ribs play an important role in lung dosimetry. Especially for low-energy radiation, the rib cage acts as a protective shield for the vital organs (such as lung and heart) in the thoracic cavity. In order to calculate the dose accurately, for each case the rib cage was changed and fitted to the new lung boundary. For this purpose, during the inflation or deflation of the lung, the rib cage was also scaled using approximately the same factor. Since there was no logical correlation between the rib thickness and the lung size, the ratio of the lung size to rib cage volume was considered as a guide. Figure 2 indicates the correlation between the size of the lung and the surrounding rib cage for constructed statistical phantom in comparison with data taken from medical images of normal patients. Accordingly, for constructed phantom, the correlation between the lung size and rib cage volume is not far from reality.

**Fig. 3.**
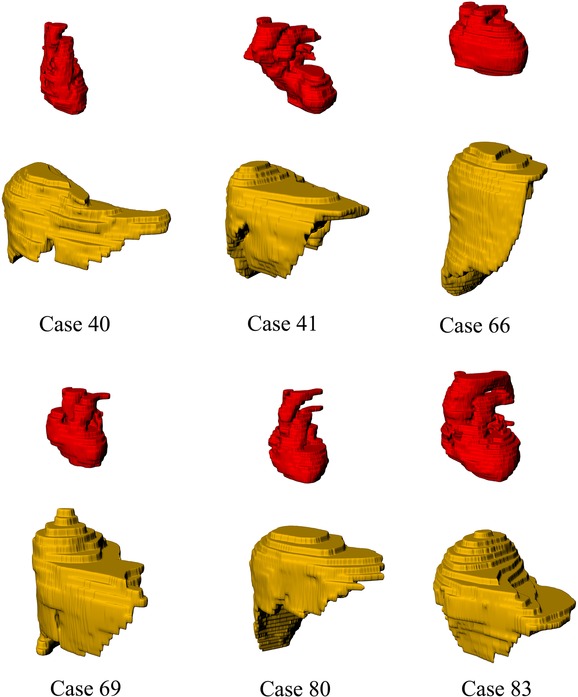
Three-dimensional models of the heart and liver of six patients who belong to the same height/weight percentile.

Other internal organs such as heart and liver had to be moved to minimize their overlaps with lung voxels. Studying the existing CT images showed that for individuals who belonged to the same height/weight percentile and had different lung volumes, the volumes of the other organs were not necessarily the same. Figure 3 shows the hearts and livers of six patients, who belong to the same height/weight percentile. (The magnification is the same for all images). As can be seen, Case 40 has a small heart and stretched liver, while Case 83 has a large heart and compact liver. This means that for individuals with the same chest size, one who has a large lung may have a smaller heart than one who has a small lung. Thus, during the inflation of the lung, the voxels of the heart and liver that overlapped with the lung voxels were ignored. Compared with the heart and liver of the AM phantom, this process resulted in a smaller heart and liver for models with large lungs. However, this process did not affect the lung dose significantly. To indicate the effects of removing some of the heart and liver voxels on the lung dose, another situation was also examined. In this situation, a new phantom (PH1) was created with a large lung, in which the overlaps between the lung and heart and liver disappeared by removing the extra voxels of lung in the heart and liver regions. The comparison between the results obtained for the PH1 phantom and those for the phantom in which some of the heart and liver voxels had been replaced by lung voxels (PH2) showed differences <3% (see Results section).

**Fig. 4.**
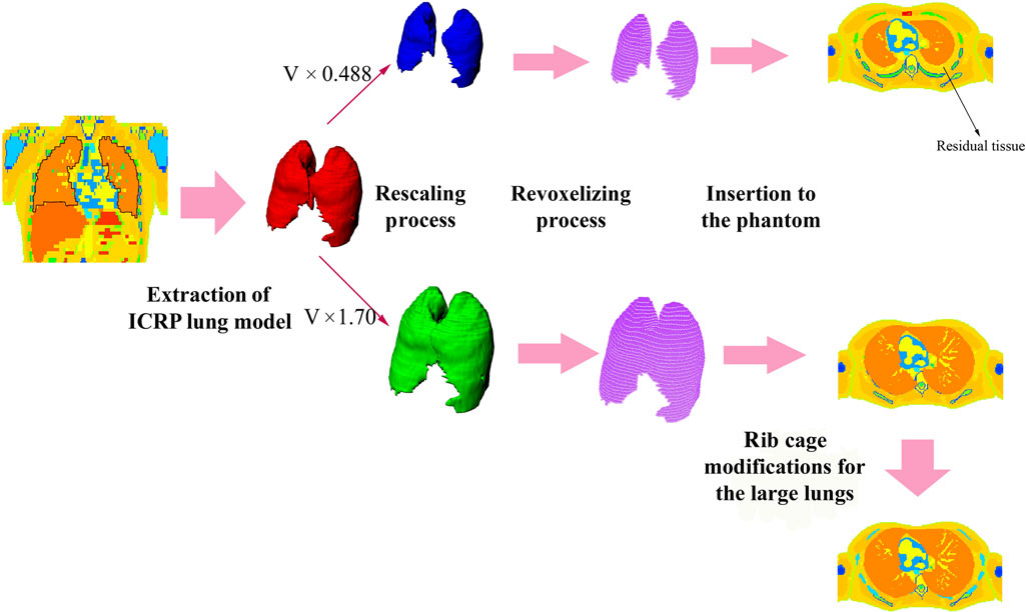
A schematic view of the development of phantoms with statistical lung masses. ICRP = International Commission on Radiological Protection; V = lung volume.

For small lungs, the size of the heart remained unchanged, because the heart and liver are soft tissues and their densities are similar to the density of residual tissue. A simple comparison showed that even replacing all of the heart and liver voxels with residual tissue did not influence the lung dose (see Results section). Consequently, the size and position of the heart and liver were fixed for phantoms with small lungs. Figure 4 shows the schematic view of the phantom construction process.

### Generation of the Gaussian mass distribution

The distribution of the lung size in a certain height/weight percentile was analyzed using IBM SPSS 20 (IBM SPSS Inc., Chicago, IL). This statistical check confirmed the normality of distribution of the lung size in a certain height/weight percentile. Accordingly, it was assumed that the distribution of the lung volume and mass followed a Gaussian distribution. Na *et al*. also made this assumption in his work published in 2010 [22].

An investigation performed on 355 Caucasoid male adults, who died for causes not related to lung deficiency or degeneration, led to a mean mass for the total lung of 1246 ± 322 g (~25% deviation from the mean value). Even considering the data of patients who did not belong to the 50th percentile, but had the same height/weight ratio, it was possible to find variability around the mean volume of the lungs of the same order. Thus, using the mean ± SD reported in ICRP89 for the 50th percentile of height/weight would be acceptable. Using a simple program, a set of 100 random numbers was generated for the target mass based on a Gaussian distribution with the mentioned mean ± SD. As aforementioned, the total mass of the lung includes blood (150.55 g) and lung tissue (1057.82 g) masses. To simplify the construction of new lungs, the resultant mass distribution was converted to a volume distribution by dividing the mass data by the relative density, which was assessed by the following equation:

(1) $$\rho_{\mathrm{re}}=\frac{\mathrm{Total lung mass}}{\mathrm{Total lung volume}}=\frac{1208.\;37\;g}{2911.\;190\;{\mathrm{cm}}^{3}}\;=\;\;0.\;4150\;g{\mathrm{cm}}^{-3}$$

### Evaluation of uncertainty parameters

In this study, the uncertainty includes two components: (i) the relative statistical uncertainty due to Monte Carlo calculations; and (ii) the standard deviation (SD) arising from the lung volume variation. Since the statistical error due to the Monte Carlo calculation is much less than that of the lung size variation, the total standard deviation would be approximately equal to the standard deviation due to the lung size variation.

(2) $$\mathrm{SD}\;=\;\sqrt{\frac{\sum {(D_{i}-\overset{®}{D})}^{2}}{N}}$$

in which i index shows the statistical phantoms number, *N* is the number of phantoms in the series, and $D_{i}$ and $\overset{®}{D}$ indicate the calculated LADF using the *i*th phantom and the average LADF, respectively.

The coefficient of variation (CV) is also used to compare the amount of LADF data deviation from the mean value for the various forms of the radiation exposure.

(3) $$\mathrm{CV}\;=\;\frac{\mathrm{SD}}{\overset{®}{D}}$$

### Monte Carlo simulation

Dosimetry calculations were performed by the Monte Carlo N-particle code version 5 (MCNP5), which simulates radiation transport inside complex structures made by different materials or tissues in the human body [23]. The cross-sections used in this study were chosen from the ENDF/B-VII libraries [24].

The verification of dose calculation was approved by comparing the organ-absorbed dose with the fluence of the AM phantom with conversion coefficients reported in ICRP publication 116 [3].

**Fig. 5.**
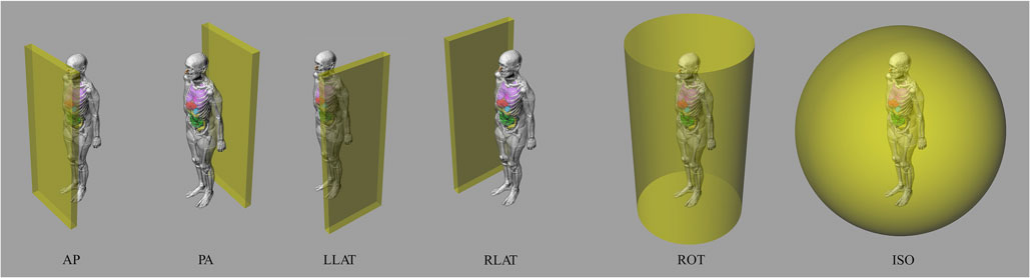
The relative position of the source and phantom in various irradiation geometries. AP = anterior–posterior irradiation, PA = posterior–anterior irradiation, LLAT and RLAT = left and right lateral axes irradiation, ROT = rotational directions around the phantom irradiation, ISO = fully isotropic irradiation.

For the simulation, unidirectional broad parallel beams along the anterior–posterior (AP), posterior–anterior (PA), left and right lateral axes (LLAT and RLAT), rotational directions around the phantom (ROT), and fully isotropic (ISO) irradiations were considered. Figure 5 shows the source specification in a schematic view.

Neutrons undergo many interactions, in which a wide range of radiations can be emitted. The types of secondary particles produced in neutron interactions are strongly energy dependent. For thermal neutrons, photons emitted during neutron capture play important roles in the organ-absorbed dose, while at energies above a few keV recoiled protons contribute to the major fraction of the absorbed dose. The mechanism of energy deposition of fast neutrons is the production of charged particles by nuclear reactions. However, at energies <20 MeV the ranges of recoiled protons and produced charged particles are very small; so that the kerma approximation is generally acceptable for the given neutron energy. Therefore, the combined MCNP5 F6:n,p tallies, which apply the kerma approximation, were employed to compute the organ-absorbed dose. For these cases, 1 × 10^7^ particle histories were simulated to achieve relative errors of <0.5%. The average execution time for each case was ~100 min.

For external photon exposures, as long as the electron equilibrium is established in the volume of interest, kerma approximation is valid. In the present work, LADFs were calculated using the kerma approximation (F6:p) for up to 500 keV photons. Above 500 keV, secondary electrons were also transported, and the energy deposition tally, *F8, with units of energy deposited per source particle was employed instead of the F6 tally. The result of the *F8 tally was divided by the mass of the cell to provide the absorbed dose. The total photon histories tracked in the Monte Carlo simulations were 2 × 10^9^ for 15 keV, 1 × 10^8^ for 20–500 keV, and 1 × 10^7^ for the higher energies. Computation time for different energies varies from 100 to 5000 min. For photon exposures, the relative standard deviations for single evaluations were small, generally <0.5% for energies >30 keV, and up to 2% for energies <30 keV. For neutron exposures, in each case, the LADF had a maximum relative error of ~0.5%.

For a better analysis of the chest region, energy deposition distribution was calculated using the track length rectangular mesh tally with the use of a tally multiplier (FM) card. The calculations were implemented for 24 mono-energetic photon sources and 19 mono-energetic neutron sources. For the simulation, a personal computer equipped with a 3.06 GHz Intel core i7 processor and 6 GB RAM, operated by Microsoft Windows 7 was used. The total execution time for all simulations reported in this work was 53 000 core-hours.

## RESULTS

First, the effects of the simplifying assumptions made about the construction of the phantoms on the obtained LADFs were examined. After that, the results obtained are presented in two separate parts. The first part consists of the assessed LADFs of the statistical phantoms for photon exposures. The second part provides the results in the presence of mono-energetic neutron sources.

### The examination of simplifying assumptions

The effects of removing some of the heart and liver voxels on the lung dose are presented in Table 2. The PH1 and PH2 were similar phantoms with large lungs. In PH1, the overlaps between the lung voxels and heart and liver voxels disappeared by removing the extra voxels of the lung, whereas in PH2 some of the heart and liver voxels were replaced by lung voxels. The results for three irradiation geometries (AP, LLAT and ISO) and three different energies (0.02, 1 and 10 MeV) showed differences of <3%.

**Table 2.** Comparison of the LADFs (in pGy cm^2^) for photons obtained using phantoms PH1 and PH2

**Phantom**	**Energy (MeV)**	**AP**	**LLAT**	**ISO**
**PH1**	**0.02**	0.187	0.025	0.050
	**1**	4.50	2.81	3.37
	**10**	22.56	20.46	20.87
**PH2**	**0.02**	0.182	0.024	0.049
	**1**	4.56	2.87	3.42
	**10**	22.22	20.60	21.46

In PH1, the overlaps between the lung voxels and heart and liver voxels disappeared by removing the extra voxels of the lung, while in PH2 some of the heart and liver voxels were replaced by lung voxels. LADF = lung-absorbed dose per fluence, AP = anterior–posterior irradiation, LLAT = left lateral axes irradiation, ISO = fully isotropic irradiation.

In Table 3, the results obtained using a phantom without heart and liver are compared with the results obtained using a phantom with heart and liver. The first phantom was created by replacing the heart and liver voxels with residual tissue voxels. It can be seen that even removing all of the heart and liver voxels did not change the lung-absorbed dose by more than 1%.

**Table 3.** Comparison of the LADFs for photons as assessed by a normal phantom (with heart and liver) with those assessed by a simplified phantom (in which the heart and liver were replaced by residual tissue)

**Phantom**	**Energy (MeV)**	**AP**	**LLAT**	**ISO**
**With heart and liver**	**0.02**	0.0671	0.0070	0.0155
	**1**	4.42	2.73	3.24
	**10**	23.89	20.56	21.52
**Without heart and liver**	**0.02**	0.0675	0.0070	0.0155
	**1**	4.41	2.70	3.20
	**10**	23.97	20.31	21.38

LADF = lung-absorbed dose per fluence, AP = anterior–posterior irradiation, LLAT = left lateral axes irradiation, ISO = fully isotropic irradiation.

### Mono-energetic photon sources

The mean ± SD values of the LADFs of statistical phantoms that were irradiated by photons are tabulated in Table 4. The mean values obtained can be compared with the reference data that were reported in ICRP Publication 116. In order to provide a comparison between the mean values and the reference datasets, the reference data are also presented in this table. Some differences between the reference data and the mean values obtained using statistical phantoms can be observed. Although the differences exceed 80% at 15 keV, because the standard deviation is of the same order as that of the mean value, the reference datum has been located in the confidence interval. By increasing the source energy, the SD decreases; and for energies between 30 and 500 keV, the reference datum is located outside the confidence interval in some cases. Above 500 keV, for LLAT and RLAT irradiation geometries, small differences can still be observed; however, for other geometries the confidence intervals contain the reference data.

**Table 4.** The LADF mean values and their SDs in comparison with the corresponding reference data (in pGy cm^2^) for broad parallel beams of mono-energetic photons

Energy (MeV)	AP		PA		LLAT		RLAT		ROT		ISO	
	Statistical data	Reference data	Statistical data	Reference data	Statistical data	Reference data	Statistical data	Reference data	Statistical data	Reference data	Statistical data	Reference data
**0.015**	0.016 ± 0.014	0.010	0.0007 ± 0.0005	0.0004	0.0014 ± 0.0017	0.0007	0.0008 ± 0.001	0.0003	0.0037 ± 0.0038	0.0021	0.0028 ± 0.0028	0.0017
**0.02**	0.086 ± 0.027	0.076	0.014 ± 0.004	0.009	0.0091 ± 0.0036	0.008	0.0075 ± 0.0029	0.005	0.027 ± 0.009	0.022	0.02 ± 0.007	0.017
**0.03**	0.245 ± 0.022	0.230	0.105 ± 0.010	0.083	0.042 ± 0.005	0.037	0.043 ± 0.005	0.037	0.113 ± 0.011	0.099	0.086 ± 0.009	0.076
**0.04**	0.309 ± 0.013	0.290	0.188 ± 0.010	0.168	0.075 ± 0.004	0.068	0.076 ± 0.004	0.068	0.173 ± 0.009	0.157	0.136 ± 0.008	0.124
**0.05**	0.336 ± 0.008	0.319	0.239 ± 0.008	0.226	0.099 ± 0.003	0.090	0.100 ± 0.004	0.091	0.206 ± 0.008	0.193	0.165 ± 0.006	0.154
**0.06**	0.357 ± 0.006	0.342	0.277 ± 0.006	0.268	0.116 ± 0.003	0.109	0.117 ± 0.003	0.109	0.233 ± 0.005	0.220	0.188 ± 0.005	0.178
**0.08**	0.412 ± 0.004	0.396	0.345 ± 0.005	0.340	0.149 ± 0.002	0.142	0.149 ± 0.003	0.141	0.281 ± 0.005	0.271	0.232 ± 0.004	0.223
**0.1**	0.484 ± 0.004	0.468	0.415 ± 0.004	0.415	0.185 ± 0.003	0.177	0.183 ± 0.003	0.176	0.334 ± 0.004	0.329	0.281 ± 0.004	0.273
**0.15**	0.709 ± 0.008	0.691	0.615 ± 0.007	0.629	0.289 ± 0.006	0.279	0.292 ± 0.007	0.278	0.509 ± 0.009	0.500	0.424 ± 0.036	0.417
**0.2**	0.957 ± 0.012	0.936	0.838 ± 0.010	0.859	0.410 ± 0.009	0.397	0.414 ± 0.010	0.395	0.698 ± 0.0132	0.688	0.588 ± 0.050	0.577
**0.3**	1.46 ± 0.02	1.43	1.29 ± 0.02	1.32	0.683 ± 0.014	0.662	0.689 ± 0.017	0.655	1.10 ± 0.02	1.08	0.937 ± 0.078	0.919
**0.4**	1.95 ± 0.02	1.91	1.77 ± 0.02	1.78	0.972 ± 0.020	0.941	0.977 ± 0.022	0.936	1.50 ± 0.03	1.48	1.29 ± 0.11	1.27
**0.5**	2.41 ± 0.03	2.38	2.21 ± 0.02	2.23	1.27 ± 0.02	1.23	1.27 ± 0.03	1.22	1.89 ± 0.03	1.87	1.65 ± 0.13	1.62
**0.6**	2.86 ± 0.03	2.82	2.65 ± 0.02	2.65	1.57 ± 0.04	1.52	1.57 ± 0.03	1.51	2.27 ± 0.04	2.25	2.00 ± 0.16	1.97
**0.8**	3.69 ± 0.04	3.64	3.45 ± 0.03	3.44	2.17 ± 0.04	2.10	2.16 ± 0.04	2.08	3.00 ± 0.05	2.98	2.65 ± 0.20	2.62
**1**	4.5 ± 0.1	4.39	4.19 ± 0.04	4.17	2.76 ± 0.04	2.67	2.75 ± 0.05	2.64	3.68 ± 0.05	3.67	3.28 ± 0.23	3.26
**1.5**	6.1 ± 0.1	6.04	5.81 ± 0.05	5.82	4.11 ± 0.05	4.01	4.10 ± 0.06	3.96	5.24 ± 0.07	5.18	4.74 ± 0.28	4.72
**2**	7.6 ± 0.1	7.47	7.20 ± 0.05	7.24	5.36 ± 0.06	5.24	5.34 ± 0.07	5.17	6.59 ± 0.08	6.52	6.03 ± 0.28	5.99
**3**	10.1 ± 0.1	9.94	9.65 ± 0.05	9.67	7.61 ± 0.07	7.45	7.52 ± 0.10	7.37	9.04 ± 0.08	8.90	8.33 ± 0.32	8.24
**4**	12.3 ± 0.1	12.1	11.9 ± 0.1	11.8	9.66 ± 0.08	9.46	9.58 ± 0.11	9.38	11.2 ± 0.1	11.0	10.4 ± 0.3	10.3
**5**	14.4 ± 0.1	14.3	13.9 ± 0.1	13.8	11.6 ± 0.1	11.4	11.5 ± 0.1	11.3	13.2 ± 0.1	13.0	12.5 ± 0.4	12.3
**6**	16.4 ± 0.1	16.2	15.9 ± 0.1	15.8	13.4 ± 0.1	13.2	13.4 ± 0.1	13.1	15.2 ± 0.1	14.9	14.3 ± 0.4	14.2
**8**	20.1 ± 0.2	20.0	19.8 ± 0.1	19.7	17.1 ± 0.1	16.8	17.0 ± 0.2	16.7	18.9 ± 0.2	18.6	18.0 ± 0.5	17.8
**10**	23.6 ± 0.3	23.4	23.8 ± 0.1	23.6	20.6 ± 0.1	20.3	20.6 ± 0.2	20.1	22.6 ± 0.1	22.3	21.6 ± 0.6	21.3

LADF = lung-absorbed dose per fluence, AP = anterior–posterior irradiation, PA = posterior–anterior irradiation, LLAT and RLAT left and right lateral axes irradiation, ROT = rotational directions around the phantom irradiation, ISO = fully isotropic irradiation.

It is also obvious from the data given in Table 4, that the variation of the LADF around the mean value is smaller for higher photon energies. This means that increasing the source energy leads to a decrease in the LADF uncertainty due to the lung size variation, so that the average LADF can be attributed to all members of the population.

**Fig. 6.**
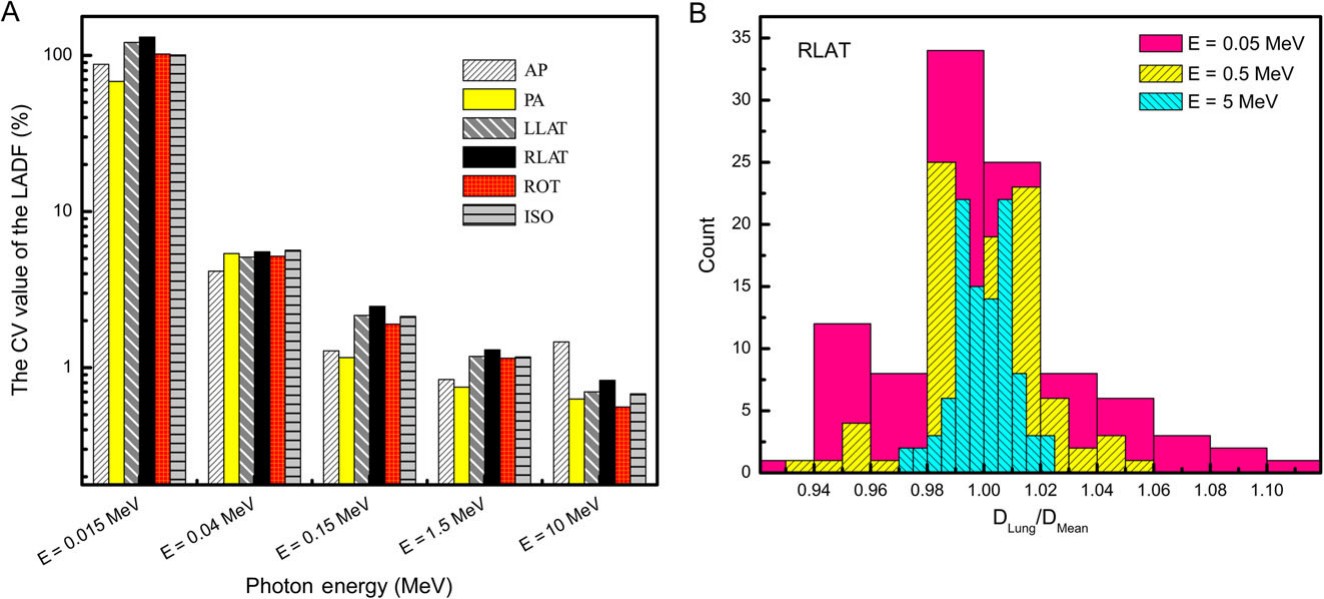
(A) Variation of the coefficient of variation (CV) value in terms of photon energy in various irradiation geometries. (B) Lung-absorbed dose per fluence (LADF) per mean value on the histogram for three different photon energies. AP = anterior–posterior irradiation, PA = posterior–anterior irradiation, LLAT and RLAT = left and right lateral axes irradiation, ROT = rotational directions around the phantom irradiation, ISO = fully isotropic irradiation.

Figure 6a provides a better demonstration of the discrepancy of the LADFs around the mean values for some energies in each irradiation geometry. For all irradiation geometries, the CV value of the LADF follows a similar pattern and greatly decreases with increasing photon energy. However, the CV value of the LADF in RLAT geometry is somewhat greater than for the other irradiation geometries, which means that the LADF is scattered to a greater extent in RLAT geometry.

In Fig. 6b, the LADF per mean value in RLAT irradiation geometry is indicated on the histogram for three different energies. At first glance, it appears that the LADF follows a Gaussian distribution, which shows the fluctuations of the LADF due to anatomical changes. These histograms are normalized, so that the width of the histogram reveals the LADF uncertainty. The large value of the LADF statistical fluctuation for 0.05 MeV means that the volume differences are essential parameters in the dose calculation. By increasing the energy, the width of the LADF distribution became smaller, and for high-energy photons it eventually became narrow. The dependency of the histogram width on the source energy indicated that for high-energy photons, which have more penetration power, the LADF fluctuation due to the anatomical changes became smaller.

### Mono-energetic neutron sources

**Fig. 7.**
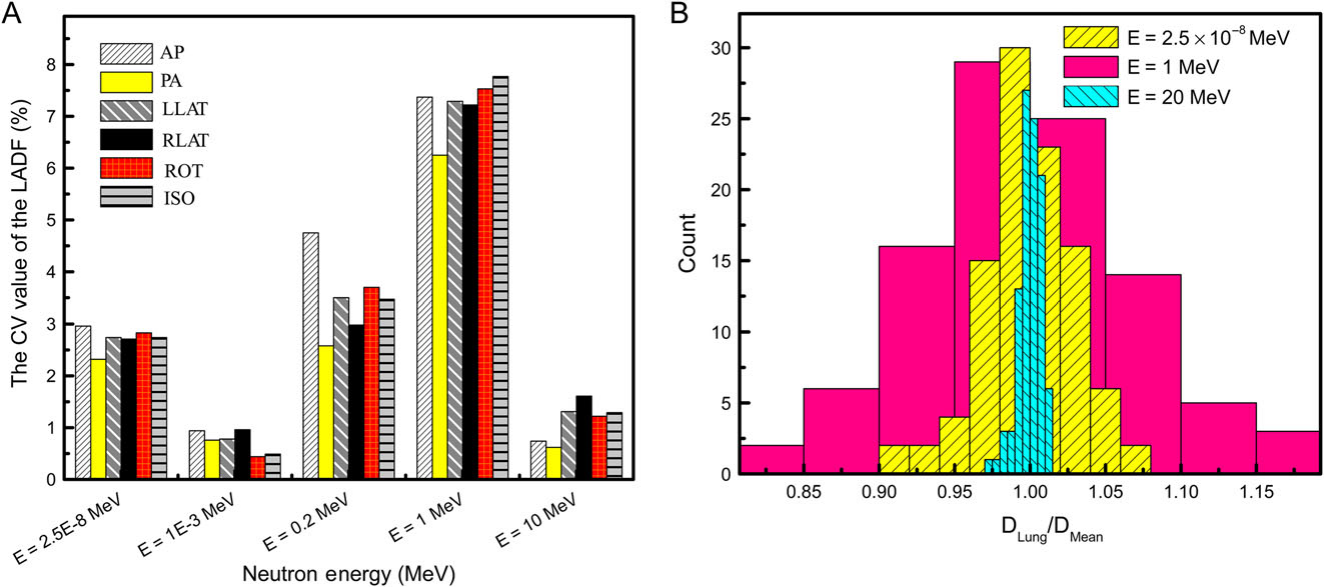
(A) Variation of the coefficient of variation (CV) value in terms of neutron energy in various irradiation geometries. (B) Lung-absorbed dose per fluence (LADF) per mean value on the histogram for three different neutron energies. AP = anterior–posterior irradiation, PA = posterior–anterior irradiation, LLAT and RLAT = left and right lateral axes irradiation, ROT = rotational directions around the phantom irradiation, ISO = fully isotropic irradiation.

Table 3 presents LADF mean values and their SDs in comparison with the reference data in the presence of neutron sources. In accordance with the data given in Table 5, there are small differences between the average LADFs and the reference data at all energies in the range of 2.5 × 10^−8^ MeV to 20 MeV. Similar to the reference data of the LADF given in Table 4, in most cases, the reference data for idealized neutron exposures lie within one standard deviation of their means. Although the LADF fluctuation in terms of the lung volume is small in total, it became somewhat significant when the neutron energy ranged from 0.1 to 5 MeV. Such behavior caused the CV values of the LADF to have a peak at ~1 MeV. Calculation of the CV value for various energies showed that the CV increased up until 1 MeV, and then fell to <2% for various irradiation geometries (see Fig. 7a). In consequence, the histogram width of the LADF per mean value, shown in Fig. 7b, tended to broaden at neutron energies of ~1 MeV. Figure 7b depicts the LADF per mean value histogram for three different energies. The high CV value is the cause of the wide-spread histogram of the LADF for 1 MeV neutrons. For energies both above and below 1 MeV, the fluctuation of the LADF due to lung mass variations became significantly smaller.

**Table 5.** The LADF mean values and their SDs in comparison with the corresponding reference data (in pGy cm^2^) for broad parallel beams of mono-energetic neutrons

**Energy (MeV)**	**AP**		**PA**		**LLAT**		**RLAT**		**ROT**		**ISO**	
	Statistical data	Reference data	Statistical data	Reference data	Statistical data	Reference data	Statistical data	Reference data	Statistical data	Reference data	Statistical data	Reference data
**2.5E-8**	1.32 ± 0.04	1.29	1.08 ± 0.03	1.14	0.407 ± 0.011	0.410	0.397 ± 0.011	0.395	0.868 ± 0.025	0.853	0.663 ± 0.018	0.665
**1E-7**	1.78 ± 0.05	1.74	1.47 ± 0.03	1.55	0.535 ± 0.014	0.528	0.517 ± 0.013	0.502	1.146 ± 0.029	1.13	0.880 ± 0.025	0.877
**1E-6**	2.57 ± 0.05	2.47	2.21 ± 0.05	2.28	0.755 ± 0.014	0.746	0.741 ± 0.015	0.721	1.67 ± 0.031	1.62	1.276 ± 0.024	1.27
**1E-5**	2.93 ± 0.03	2.83	2.57 ± 0.04	2.64	0.861 ± 0.015	0.843	0.852 ± 0.015	0.819	1.909 ± 0.027	1.86	1.469 ± 0.022	1.45
**1E-4**	2.96 ± 0.02	2.86	2.65 ± 0.03	2.74	0.881 ± 0.011	0.867	0.867 ± 0.011	0.842	1.937 ± 0.020	1.91	1.511 ± 0.012	1.48
**1E-3**	2.90 ± 0.03	2.82	2.65 ± 0.02	2.72	0.870 ± 0.007	0.861	0.859 ± 0.008	0.839	1.927 ± 0.009	1.90	1.508 ± 0.007	1.48
**1E-2**	2.85 ± 0.03	2.79	2.62 ± 0.02	2.73	0.865 ± 0.004	0.848	0.857 ± 0.006	0.842	1.910 ± 0.009	1.88	1.492 ± 0.008	1.47
**0.02**	2.92 ± 0.03	2.82	2.70 ± 0.02	2.75	0.887 ± 0.006	0.865	0.852 ± 0.006	0.840	1.94 ± 0.01	1.90	1.508 ± 0.008	1.49
**0.05**	3.06 ± 0.02	2.97	2.80 ± 0.02	2.87	0.927 ± 0.008	0.907	0.892 ± 0.007	0.862	2.05 ± 0.01	1.99	1.572 ± 0.008	1.56
**0.1**	3.40 ± 0.08	3.29	3.01 ± 0.03	3.08	0.988 ± 0.019	0.965	0.960 ± 0.016	0.923	2.20 ± 0.04	2.16	1.720 ± 0.025	1.69
**0.2**	4.27 ± 0.20	4.10	3.50 ± 0.09	3.61	1.16 ± 0.04	1.12	1.12 ± 0.03	1.08	2.61 ± 0.10	2.58	2.04 ± 0.07	2.01
**0.5**	7.26 ± 0.50	6.95	5.43 ± 0.30	5.62	1.76 ± 0.10	1.69	1.75 ± 0.10	1.65	4.19 ± 0.28	4.12	3.25 ± 0.22	3.20
**1**	10.96 ± 0.80	11.6	7.87 ± 0.49	9.36	2.60 ± 0.18	2.85	2.61 ± 0.18	2.87	6.31 ± 0.47	7.00	4.89 ± 0.38	5.51
**2**	21.35 ± 0.85	20.6	17.7 ± 0.8	17.5	6.47 ± 0.33	6.10	6.60 ± 0.35	6.15	13.80 ± 0.67	13.3	11.12 ± 0.58	10.7
**3**	28.42 ± 0.78	27.8	24.6 ± 0.7	24.6	10.17 ± 0.41	9.56	10.32 ± 0.43	9.63	19.62 ± 0.71	18.9	16.23 ± 0.65	15.7
**5**	39.42 ± 0.83	38.5	35.1 ± 0.8	35.3	17.13 ± 0.55	16.0	17.39 ± 0.59	16.1	28.73 ± 0.83	28.2	24.79 ± 0.79	23.9
**10**	55.16 ± 0.73	54.4	50.5 ± 0.6	51.4	29.21 ± 0.59	28.2	29.10 ± 0.67	28.0	43.68 ± 0.79	43.2	37.69 ± 0.82	37.7
**14**	63.79 ± 0.57	62.3	59.6 ± 0.6	59.2	36.19 ± 0.63	35.1	1.79 ± 0.10	34.8	52.20 ± 0.79	50.7	45.09 ± 0.81	45.0
**20**	73.05 ± 0.54	68.7	69.4 ± 0.4	66.0	45.50 ± 0.60	42.9	45.04 ± 0.73	42.5	61.50 ± 0.75	58.0	54.63 ± 0.70	52.2

LADF = lung-absorbed dose per fluence, AP = anterior–posterior irradiation, PA = posterior–anterior irradiation, LLAT and RLAT left and right lateral axes irradiation, ROT = rotational directions around the phantom irradiation, ISO = fully isotropic irradiation.

**Fig. 8.**
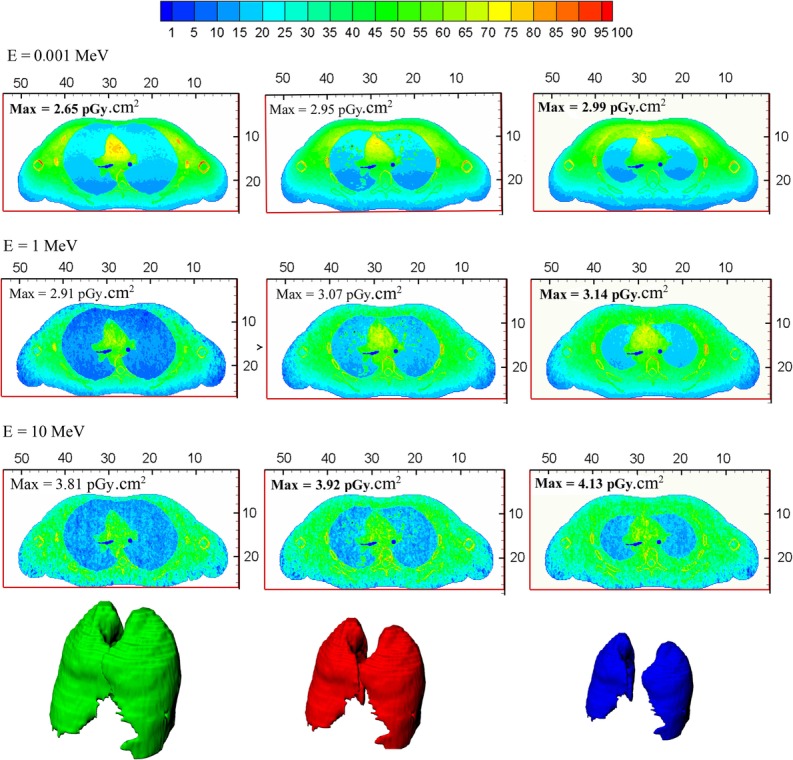
Deposited energy distribution due to secondary photons in anterior–posterior irradiation. Three different neutron energies (0.001, 1 and 10 MeV) are considered here. The plots in the first column belong to the phantom with the largest lung. The third column shows the plots of the phantom into which the smallest lung has been imported. Finally, the middle column corresponds to the ICRP phantom with the normal lung. The lung in each particular row are known to have been irradiated by incident neutrons of similar energy.

**Fig. 9.**
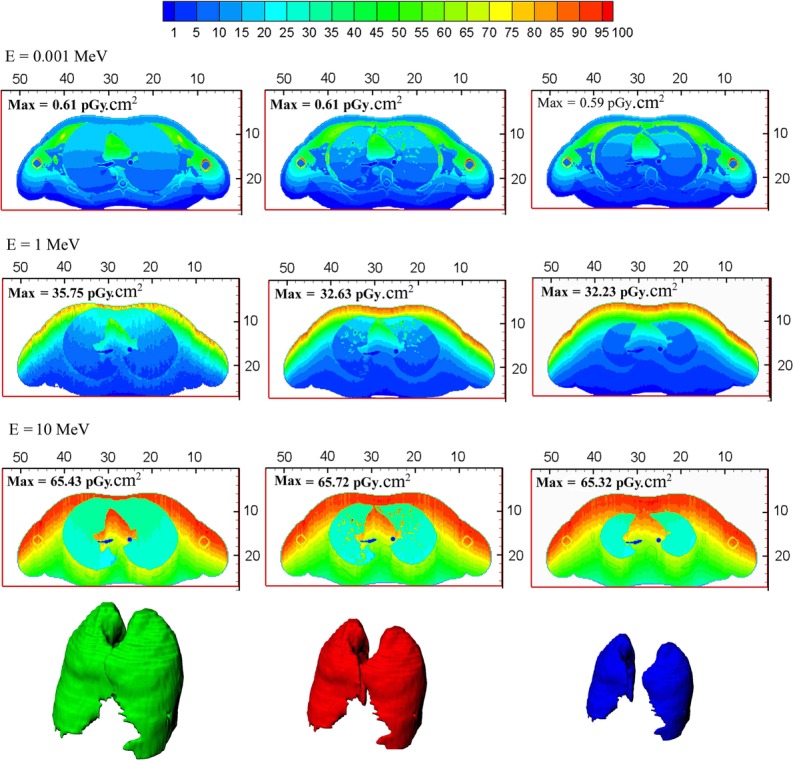
Deposited energy distribution due to recoiled protons in anterior–posterior irradiation. Three different neutron energies (0.001, 1 and 10 MeV) are considered here. The plots in the first column belong to the phantom with the largest lung. The third column shows the plots of the phantom into which the smallest lung has been imported. Finally, the middle column corresponds to the ICRP phantom with the normal lung. Images in each particular row had similar conditions in terms of the energy of the incident neutrons.

In order to explain the variation in the CV value in terms of source energy, the details of the neutron energy deposition in the thorax were studied. Figures 8 and 9 depict cross-sectional plots of the energy deposition in the thorax region for three phantoms into which the largest, smallest and intermediate lungs were imported. In Fig. 8, the absorbed dose contribution due to the photons is indicated, whereas Fig. 9 reveals the proton contribution to the absorbed dose. The plots are provided for AP irradiation and three different neutron energies (0.001, 1, and 10 MeV). The first and third columns illustrate phantoms with the largest and smallest lungs. However, plots in the middle column belong to the phantom with the ICRP lung. Images in each particular row had similar conditions in terms of the energy of the incident neutrons.

In order to simplify comparisons between the plotted results, the deposed energies were normalized to their maximum amount, so the legend expresses the percentage of the maximum value of the deposited dose. The uniformity of the distribution energy deposition due to secondary photons is evident from Fig. 8, except for 0.001 MeV neutrons. This means that the delivered dose to the lung may not be influenced by the lung size. In the case of 0.001 MeV neutrons, in spite of the non-uniformity of the energy deposition distribution in the lung, there was no significant difference between the total doses received by lungs of different sizes. However, the contribution of the neutron-induced protons to the energy deposition in the lungs strongly depended on the incident neutron energy. It can be seen from Fig. 9 that for low-energy neutrons, secondary protons deposited their energy in superficial layers of the body. By increasing the neutron energy, a larger part of the lung was exposed to recoiled protons.

## DISCUSSION

Although there are several sets of ICRP-compatible phantoms, they are all based on person-specific anatomical data. However, using reference AM and female ICRP voxel phantoms, one can improve the accuracy of dose estimation. The reference conversion coefficients assessed by ICRP phantoms were provided for general use in radiological protection practice for occupational exposures. This work is a primary study to quantify the significance of dosimetric uncertainty due to the variation in the lung size. Since the reference data are the values given for a reference phantom, actually they cannot be used for individual cases. We have sought to provide some data in Table 4 and Table 5 that could be employed to give an idea of the variability of the reference data for individual examinations. To highlight the dependency of dosimetric uncertainty on the incident photon energy, the mean values of the LADF, together with their SDs and their corresponding reference data are shown in Table 4. The mean values reported in this paper were obtained by averaging the LADF values from 100 phantoms with different lung sizes.

It is expected that the mean values assessed by a population of statistical phantoms would have good agreement with the reference data obtained from the ICRP/ICRU adult voxel model. However, there are some differences between the mean values and the reference data. For 15 keV photons, the difference between the average LADF and the reference data is >80%; however, the SD of the mean value is significantly large; so that the reference data is located within the confidence interval. For photon energies of between 30 and 500 keV, the confidence interval becomes smaller, and differences between the average LADF and the reference data become more meaningful. The observed differences can be explained by detailed review of the LADF amounts for statistical phantoms. Comparison of the LADF values showed that halving the volume of the reference lung led to a decrease in the delivered dose to the lung of between 2.5- and 4-fold, but doubling the volume increased the lung-absorbed dose by a factor of between 10 and 23. Table 6 shows the LADF for three phantoms exposed to 15 keV photons. The lung volume of the first phantom (Sample 1) was about half of the lung volume of the second phantom (Sample 2). Moreover, the lung volume of the third phantom (Sample 3) was slightly less than twice the lung volume of the second phantom.

**Table 6.** Comparison of the LADF (in pGy cm^2^) of three phantoms with different lung volumes for six standard irradiation geometries

**Phantom**	**AP**	**PA**	**LLAT**	**RLAT**	**ROT**	**ISO**
**Sample 1**	0.0024	0.0001	0.0002	0.0001	0.0004	0.0004
**Sample 2**	0.0077	0.0005	0.0005	0.0003	0.0017	0.0013
**Sample 3**	0.0792	0.0029	0.01	0.0071	0.0229	0.0172

LADF = lung-absorbed dose per fluence, AP = anterior–posterior irradiation, PA = posterior–anterior irradiation, LLAT and RLAT left and right lateral axes irradiation, ROT = rotational directions around the phantom irradiation, ISO = fully isotropic irradiation.

For weakly penetrating photons, the superficial layers of the body (including muscle, residual tissue and ribs) are strong barriers that prevent photons reaching the lung region. In the case of the normal lung, some photons can get through these barriers and reach the lung, but the lung-absorbed dose is generally small in the presence of low-energy photons. Because only a limited number of photons can deposit their energies in the lung, decreasing the lung size did not reduce the amount of the dose in comparison with the dose received by the normal lung. When the lung size increased, the thickness of the muscle placed in front of the lung became thin, so that a large number of low-energy photons could deposit their energies in the lung. This means that the arithmetic mean of the LADF would shift slightly from the reference data to higher values. However, above 500 keV, this difference would become negligible, and the statistical distributions of the LADF would be a symmetric distribution around the mean value.

The SDs given in Table 4 imply that increasing the source energy decreases the dependency of the LADF on the lung volume. Deviations of the mean values originate from variations in lung masses and volumes; thus, a small SD reveals a low dependency of the LADF on anatomical changes. Accordingly, in the range of 15 keV to 0.1 MeV, the LADF strongly depended on the lung volume; however, for higher energies, regardless of the lung volume, the results converged nicely to a single dose value.

The penetration power of the photon, in combination with variation in the thickness of the superficial layer located in front of the lung, can also explain the relationship between the incident energy and the SD of the mean value. For low-energy photons, which deposit most of their energies in the frontal layer of the body, the lung volume is a crucial parameter dictating the magnitude of the absorbed dose. By increasing the photon energy, a greater volume is affected by the radiation, so the deposited energy in the lung increases. Above 100 keV, the entire lung was exposed to the incident radiation, and the LADF remained approximately the same.

Another indication of the close relationship between dose uncertainty and the incident energy is provided in Fig. 6a. It can be seen that for 15 keV photons, the CV values of the LADF for all studied irradiation geometries were ~100%. Above 15 keV, the CV values of the LADF dropped significantly from 100% (to <10% for 0.04 MeV), although for higher energies, they gradually fell to ~1%. Figure 6b presents the LADF per mean value on the histogram for three different energies, in RLAT irradiation geometry. It is clear that the increasing increments of photon energy led to narrowing of the histogram width. This means that the dose uncertainty would be smaller for higher energies. It is also worth mentioning that the LADF variation for low-energy photons was too high, so the effect of eliminating internal tissues of the lung was negligible.

Neutron exposures provide another pattern for dose uncertainty. In contrast with a photon, a neutron undergoes many interactions in the human body. Due to a huge percentage of hydrogen in the body, even thermal neutrons are strongly scattered when they enter the body. Because of this, neutrons can interact in the entire lung volume, and so the dependency of the dose on the lung volume would decrease. Table 5 suggests that for a neutron exposure, regardless of its energy, the lung-absorbed dose was somewhat independent of the lung volume. Moreover, remarkable differences between the LADFs and the reference data were not observed. Although the discrepancy of the LADFs around the mean value was <10% for all energies, for very low energies and within the range of intermediate energies some deviations from mean values were evident. Figure 7a depicts the variations in the CV values in terms of the incident energy for various irradiation geometries. For all exposure situations, the CV value increased with increasing energy and hit its peak at 1 MeV. After that, it fell, reaching ~2% for the situation in which the source was emitting 10 MeV neutrons.

To explain the behavior of the CV as a function of the incident energy, the process of neutron energy deposition should be studied. The deposition of neutron energy is a complex process. For low-energy neutrons, secondary photons contribute the major fraction of the absorbed dose. Generally, neutron absorption in hydrogenated materials (such as the human body) produces a 2 MeV photon. Using kerma approximation, this photon is absorbed locally, so the lung variation does not affect the evaluated doses significantly. Therefore, the dispersion of LADF for low-energy neutrons is slightly higher than 1%. By incrementing neutron energies, not only does the range of the produced photons increase, but the contribution of these particles to the total absorbed dose becomes smaller. Figure 8 indicates the distribution of the deposited energy due to the neutron-induced photon for three different phantoms. The directions of the incidence neutrons are anterior–posterior, and the results are plotted for neutron exposures with energies of 0.001, 1 and 10 MeV.

At energies of >1 keV, the neutron energy is deposited by recoiling protons from the neutron's elastic scattering by hydrogen. However, the range of the produced proton is too short to play a role in the lung-absorbed dose. Thus, the CV value falls to <1% and it remains at almost the same level up to 0.05 MeV. For higher energies of the incident neutrons, the amount of energy transferred to the protons increases, so that some protons can liberate their energies in the lung region. Under these conditions, the organ volume becomes an important parameter for determining the proton contribution in the organ-absorbed dose. For 1 MeV neutrons, at which energy the CV value hits its peak, 80% of the absorbed dose originates from recoiled protons. As the incident energy of the neutrons increases, the proton range becomes larger, and a relatively uniform distribution of the energy deposition is provided for the lung. Therefore, the dependency of dose on organ volume decreases. This is the reason why CV values have a declining tendency with higher energies. Figure 9 indicates the contribution of the recoiled protons to the energy deposited in the lung. The short range of the produced protons and the dependency of the LADF on the lung size for 1 MeV neutrons are clearly evident.

## CONCLUSION

In the present study, the authors aimed to examine the feasibility of determining the lung absorbed dose uncertainty due to lung volume changes using the voxel phantom. For this purpose, a series of phantoms differing in lung volume were constructed, based on the AM phantom. LADFs were obtained for mono-energetic photon and neutron exposures in idealized irradiation geometries, and the average doses and their deviations were quantified for each irradiation.

The results obtained implied that for low-energy photon sources, the relationship between absorbed dose and organ volume was significant, whereas for higher energy photon sources, the organ-absorbed dose became independent of the organ volume.

In the case of neutron exposure, the lung-absorbed dose had a maximum dispersion of ~8% for LADF in the range of 0.1 to 5 MeV. For other energies, the results obtained demonstrate that the size of the lung did not have a significant impact on the absorbed dose. Although our results were restricted to cases in which the phantom was irradiated by a broad mono-energetic beam, they may be valid for diagnostic systems such as IVNAA. It is worth mentioning that in addition to the lung-absorbed dose, the lung size can affect the detected spectrum; so accurate estimation of body composition is not possible without considering the influence of the organ size.
